# Robotic‐assisted colectomy for right‐sided colon cancer: Short‐term surgical outcomes of a multi‐institutional prospective cohort study in Japan

**DOI:** 10.1002/ags3.12694

**Published:** 2023-05-23

**Authors:** Shinichi Yamauchi, Akio Shiomi, Chu Matsuda, Ichiro Takemasa, Tsunekazu Hanai, Mamoru Uemura, Yusuke Kinugasa

**Affiliations:** ^1^ Department of Gastrointestinal Surgery Tokyo Medical and Dental University Tokyo Japan; ^2^ Division of Colon and Rectal Surgery Shizuoka Cancer Center Shizuoka Japan; ^3^ Department of Gastroenterological Surgery Osaka International Cancer Institute Osaka Japan; ^4^ Department of Surgery, Surgical Oncology and Science Sapporo Medical University Hokkaido Japan; ^5^ Department of Gastroenterological Surgery Fujita Health University Aichi Japan; ^6^ Department of Gastroenterological Surgery, Graduate School of Medicine Osaka University Osaka Japan

**Keywords:** colectomy, colon cancer, laparoscopy, minimally invasive surgical procedures, robotics

## Abstract

**Background:**

In Japan, there are no substantial reports on robotic‐assisted colectomy because few institutions performed the procedure, as it was not covered by national insurance until March 2022.

**Aim:**

This study aimed to evaluate the safety and feasibility of robotic‐assisted colectomy for patients with curatively resectable colon cancer in Japan.

**Methods:**

This multi‐institutional, prospective, single‐arm, observational study enrolled patients diagnosed with curatively resectable clinical stage I–IIIC colon adenocarcinoma with D2 or D3 lymph node dissection and treated with robotic‐assisted colectomy. The primary endpoint was the conversion rate to laparotomy. The non‐inferiority of outcomes for robotic‐assisted colectomy versus laparoscopic colectomy, which was determined from historical data, was verified.

**Results:**

One hundred patients were registered between July 2019 and March 2022 and underwent robotic‐assisted colectomy performed by seven expert surgeons at six institutions. Thirteen patients were excluded because their surgeons had insufficient experience performing robotic‐assisted colectomy; therefore, 87 patients were eligible for the primary endpoint analysis. There was no conversion in these 87 patients, and robotic‐assisted colectomy was non‐inferior to laparoscopic colectomy in terms of conversion rate (90% confidence interval 0–3.38, *p* = 0.0006). No intraoperative adverse events occurred, and no mortality was observed in a total of 100 patients. The rate of patients with Clavien–Dindo complications grade III or higher was 4%.

**Conclusion:**

This study showed the non‐inferiority of the conversion rates between robotic‐assisted colectomy and laparoscopic colectomy. Favorable perioperative outcomes also suggest the safety and feasibility of robotic‐assisted colectomy.

## Graphical abstract

This multi‐institutional, prospective, single‐arm, observational study enrolled patients diagnosed with curatively resectable clinical stage I–IIIC colon cancer and treated with robotic‐assisted colectomy. The primary endpoint was the conversion rate to laparotomy. One hundred patients were registered and underwent robotic‐assisted colectomy performed in the study.

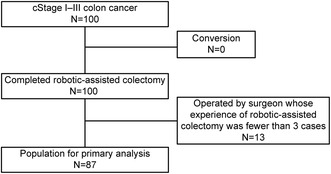

## 1 INTRODUCTION

Approximately 1.8 million new cases of colorectal cancer are diagnosed annually worldwide, and the number of annual deaths is approximately 880 000, second only to lung cancer.^1^ Asia accounts for approximately half of all new cases and deaths worldwide. The dramatic increase in colorectal cancer incidence in Asia is a rising public health concern. The incidence of this condition is increasing in Japan as well. Colorectal cancer has become an important disease in recent years, accounting for the third most common cause of cancer‐related mortality and the most common cause of morbidity.^2^, ^3^ Therefore, improving treatment outcomes for colorectal cancer is an important issue.

Surgical resection is the basis of colorectal cancer treatment. The usefulness of minimally invasive surgery (MIS) has recently been examined. Since the first report of laparoscopic colectomy (LC) in 1990,^4^ the use of laparoscopic surgery for colorectal cancer has spread rapidly. Many researchers have demonstrated the benefits of LC compared with open laparotomy, including early recovery of postoperative bowel motility and shorter hospital stay.^5^, ^6^, ^7^, ^8^, ^9^ However, challenges and limitations of laparoscopic surgery have also been observed, such as the high incidence of laparoscopic conversion to laparotomy and the lack of evidence of superiority or non‐inferiority of laparoscopic surgery with respect to long‐term outcomes such as overall survival (OS), as demonstrated in several large randomized controlled trials (RCT).^6^, ^7^, ^8^, ^10^, ^11^

Robotic‐assisted surgery (RAS) using a surgical robot is expected to overcome the disadvantages of laparoscopy because of features not found in conventional laparoscopic surgery, such as forceps with multiple joints and a wide range of motion, an anti‐shake mechanism, fine movements with motion scales, 3D high‐definition images, and image stabilization. These features enable more accurate and detailed surgery with less invasiveness and more freedom in the form of the surgeon.^12^ RAS has become popular worldwide since robotic‐assisted total prostatectomy was first reported in 2001.^13^ It is also rapidly gaining popularity for both colon and rectal cancer surgeries.^13^, ^14^ In Japan, RAS for rectal cancer has been covered by insurance since April 2018. Consequently, the number of procedures performed in Japan has rapidly increased.^15^ Recently, many studies performed in Japan have indicated the usefulness of robotic‐assisted rectal resection. On the other hand, robotic‐assisted colectomy (RAC) for colon cancer was not covered by insurance until March 2022. Until then, it had been performed at a few institutions at patients' own expenses. Thus, no substantial research on RAC has been conducted in Japan.^16^

Therefore, we designed and conducted a prospective registry study of RAC for colon cancer involving institutions in Japan that began introducing the procedure at the expense of the patients. This study aimed to evaluate the safety and feasibility of RAC for patients with stage I–III colon cancer, which could be curatively resectable.

## 2 METHODS

### 2.1 Study design

This multi‐institutional, prospective, single‐arm, open‐label, observational study evaluated the safety and feasibility of RAC for resectable colon cancer. We verified the non‐inferiority of surgical outcomes for RAC versus LC using historical data. Six institutions offering RAC at the expense of the patients who participated in this study.

Eligible patients were diagnosed with curatively resectable cStage I–IIIC (T‐14b, N0‐2b, M0) colon cancer with D2 or D3 lymph node dissection. Other selection criteria were as follows: (1) age between 20 and 80 years; (2) Eastern Cooperative Oncology Group performance status (PS) score from 0 to 2; (3) histologically diagnosed adenocarcinoma; (4) tumor located at the cecum, ascending colon, or transverse colon; (5) not indicated for endoscopic resection; (6) maximum tumor diameter less than 8 cm; (7) no multiple lesions requiring two or more locations for anastomosis; and (8) consent for medical treatment at their own expense and for participation in this research. Patients were excluded who had prior chemotherapy or radiation therapy, prior gastrectomy or bowel resection other than appendectomy, and laboratory values within 14 days prior to registration that met the following criteria: white blood cell count <3000/mm^3^, platelet count <100 000/mm^3^, hemoglobin <7.0 g/dL, AST >100 IU/L, ALT >100 IU/L, and serum Cr >1.5 mg/dL. Patients were registered from July 2019 to March 2022.

The primary endpoint was the rate of conversion to open surgery, which has been reported to be related to postoperative complications, mortality, increased blood transfusions, and recurrence due to residual cancer. The secondary endpoints included the rate of conversion among all patients who completed the protocol treatment, postoperative complication rate within 30 days, postoperative readmission rate within 30 days, surgery‐related mortality rate, intraoperative adverse event rate, postoperative recovery, surgical results, surgeon learning curve, incidence of abdominal incisional hernia within 5 years after surgery, incidence of peritoneal dissemination within 5 years after surgery, OS (5 years), and relapse‐free survival (RFS, 5 years).

For historical data on the primary endpoint, we used data from the laparoscopic group of the “Randomized Controlled Trial to Evaluate Laparoscopic versus Open Surgery for Colorectal Cancer (Japanese Clinical Oncology Group, JCOG0404),” a multicenter RCT in Japan. The clinical hypothesis of this study was that “the conversion rate of RAC would be no more than 2.7% inferior to the rate of LC (5.4% in JCOG0404),” and we decided to judge it clinically insignificant when the upper limit of the confidence interval of the conversion rate of RAC was 8.1% or higher.^11^, ^17^, ^18^ We calculated the number of patients required for analysis using an accurate method based on the binomial distribution when the expected rate of RAC was 2.0%, the one‐sided significance level was 0.05, and the power was 0.8. The number of patients required for analysis was set to 94, and the target number of patients was 94.

Standard treatment of this study was a right hemicolectomy or right colectomy with D2 or D3 lymph node dissection, which was performed with robotic assistance; however, the protocol treatment was discontinued if the colon cancer was diagnosed as unresectable by preoperative or intraoperative diagnosis. If curative resection was deemed difficult under robotic surgery, the surgery was switched to laparotomy, and the protocol treatment was performed. The surgeons were board‐certified in gastroenterology by the Japanese Society of Gastroenterological Surgery, certified by the Japan Society for Endoscopic Surgery, had a da Vinci Certificate from Intuitive Surgical, and had experience performing at least 50 cases of RAS. Under these regulations, seven expert surgeons in six institutions participated as console surgeons in this study. Surgical cases performed by surgeons with less than three cases of experience in RAC were excluded from the primary endpoint analysis population. The reason for the exclusion criteria was that most surgeons needed to get used to RAC due to their little or no experience with the procedure, although they were experts in RAS for rectal cancer.

This project was approved by the Tokyo Medical and Dental University Certified Review Board (approval number, NR2019‐001) and registered in the Japan Registry of Clinical Trials. The registration number was jRCT1032190036 (https://jrct.niph.go.jp/latest‐detail/jRCT1032190036).

### 2.2 Statistical analysis

For the primary endpoint, the number of conversion cases and incidence rate of the analyzed population were calculated, and 90% confidence intervals were estimated. The null hypothesis was tested using binomial distribution (one‐sided *α* = 0.05). For the secondary endpoints, categorical data of the cases and the rate in the cohort were calculated, and 95% confidence intervals were estimated. For continuous data, summary statistics were calculated, and 95% confidence intervals were estimated. Survival curves were estimated using the Kaplan–Meier method, and 95% confidence intervals for the median value and annual survival rate were estimated. A learning curve was prepared based on the number of surgical experiences on the horizontal axis and time of surgery on the vertical axis. *p* < 0.05 was considered statistically significant. All statistical analyses were performed using SAS software (version 9.4; SAS Institute).

## 3 RESULTS

### 3.1 Patient characteristics

One hundred patients were enrolled in this study between July 2019 and March 2022.

**FIGURE 1:**
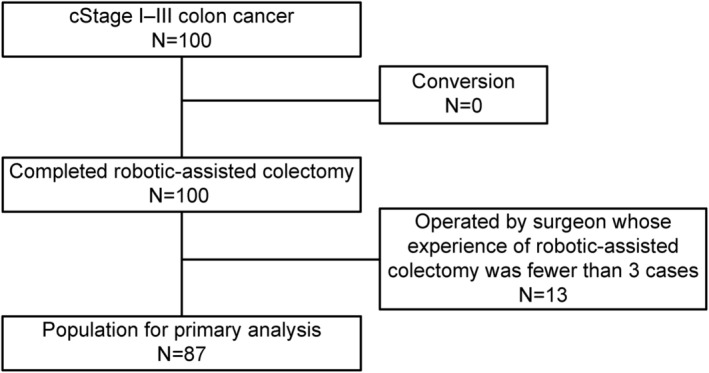
Flowchart of patient enrollment. A total of 100 patients were registered in this study. All patients were treated according to the protocol and underwent robotic‐assisted colectomy. Thirteen of these patients were excluded because their surgeons had insufficient experience performing robotic‐assisted colectomy, making a total of 87 patients eligible for the primary endpoint analysis.

All patients were treated according to the protocol, and RAC was completed. Thirteen of the 100 patients underwent surgery by surgeons with less than three cases of experience of RAC, making a total of 87 patients eligible for the primary endpoint analysis (Figure 1).

The backgrounds of the 100 patients and the 87 patients for primary endpoint analysis included in this study are shown in Table 1. The cohort of all 100 patients was in good general condition, with a median age of 69 years, median body mass index of 22.8 kg/m^2^, with 94% having a PS score of 0, of which 76% had comorbidities. Thirty‐six percent of the patients had a history of laparotomy. Regarding the percentage of the main tumor site, 22% were in the cecum, 64% in the ascending colon, and 14% in the transverse colon, with clinical stages I, II, and III in 44%, 18%, and 38% of patients, respectively (Table 2).

**TABLE 1:** Patient characteristics.

	All patients		Patients for primary analysis	
	*N* = 100	%	*N* = 87	%
Age, years (IQR)	69 (61.5–74)		69 (61–73)	
Sex				
Male	49	49	46	53
Female	51	51	41	47
BMI, kg/m^2^ (IQR)	22.8 (20.6–25.2)			
ECOG PS				
0	94	94	85	98
1	6	6	2	2
2	0	0	0	0
ASA‐PS				
Class I	26	26	24	28
Class II	73	73	62	71
Class III	1	1	1	1
Class IV	0	0	0	0
Comorbidity				
Absent	24	24	20	23
Present	76	76	67	77
Past abdominal surgical history^a^				
Absent	64	64	56	64
Present	36	36	31	36

*Note*: Data are presented as number or median.

Abbreviations: ASA‐PS, American Society of Anesthesiologists physical status; BMI, body mass index; ECOG PS, Eastern Cooperative Oncology Group performance status; IQR, interquartile range.

^a^ Without gastrectomy or intestinal resection.

**TABLE 2:** Preoperative diagnosis.

	All patients		Patients for primary analysis	
	*N* = 100	%	*N* = 87	%
Tumor location				
Cecum	22	22	18	21
Ascending	64	64	57	65
Transverse	14	14	12	14
Depth of invasion (cT)				
T1	25	25	21	24
T2	22	22	20	23
T3	33	33	29	33
T4	20	20	17	20
Lymph node metastasis (cN)				
N0	62	62	53	61
N1	22	22	19	22
N2	16	16	15	17
Distant metastasis (cM)				
M0	100	100	87	100
M1	0	0	0	0
cStage				
I	44	44	38	44
II	18	18	15	17
III	38	38	34	49
IV	0	0	0	0
Multiple cancer				
Absent	96	96	83	95
Present	4	4	4	5
Double cancer				
Absent	99	99	86	99
Present	1^a^	1	1^a^	1

*Note*: Data are presented as numbers.

^a^ Right renal cell carcinoma.

### 3.2 Surgical outcomes

Details of the surgery and short‐term surgical outcomes are shown in Tables 3 and 4. Eighty‐nine percent of the patients underwent intracorporeal anastomosis and 91% underwent D3 dissection. No patients underwent conversion to laparotomy, and no intraoperative adverse events occurred. The first flatus was confirmed on postoperative day 3, and the length of postoperative hospital stay was 6 days. The rate of patients with complications of Clavien–Dindo Classification Grade II or higher was 11%, but Grade III complications were observed in only 4% of patients. The readmission rate within 30 days after the operation was 2%. None of the patients died.

**TABLE 3:** Surgical procedure and intraoperative surgical outcomes.

	All patients		Patients for primary analysis	
	*N* = 100	%	*N* = 87	%
Type of surgery				
Right hemi‐colectomy	79	79	69	79
Right colectomy	21	21	18	21
Anastomosis				
Intracorporeal	89	89	78	90
Extracorporeal	11	11	9	10
Lymph node dissection				
D2	9	9	9	10
D3	91	91	78	90
Harvested lymph node (IQR)	33 (25–41)		33 (26–41)	
Resection of adjacent structures				
Absent	99	99	86	99
Present	1^a^	1	1^a^	1
Conversion				
Absent	100	100	87	100
Present	0	0	0	0
Intraoperative complication				
Absent	100	100	87	100
Present	0	0	0	0
Operative time, minutes (IQR)				
Total	211 (178.5–262)		210 (175–263)	
Time on console	160 (126.5–197)		159 (127–198)	
Time for anastomosis^b^	30.5 (25–38.5)		29 (25–38)	
Blood loss, mL (IQR)	0 (0–5)		0 (0–5)	
Transfusion				
Absent	99	99	86	99
Present	1	1	1	1

*Note*: Data are presented as number or median.

Abbreviation: IQR, interquartile range.

^a^ Abdominal wall and gonadal vessels.

^b^ Time from intestinal resection of both oral and anal side to completion of anastomosis.

**TABLE 4:** Postoperative course and complications.

	All patients		Patients for primary analysis	
	*N* = 100	%	*N* = 87	%
Postoperative hospital stay, day (IQR)	6 (5–8)		6 (5–8)	
Days to the first flatus, day (IQR)	3 (2–3)		3 (2–3)	
Days to soft diet, day (IQR)	3 (3–3)		3 (3–3)	
Postoperative complication (C–D > grade II)				
Total: grade II/grade III	7/4	7/4	6/3	7/3
Surgical site infection	3	3	2	2
Ileus	2	2	2	2
Bowel obstruction	0	0	0	0
Anastomotic leakage	0	0	0	0
Anastomotic stenosis	1	1	1	1
Anastomotic bleeding	1	1	0	0
Others	4	4	4	5
Re‐admission within 30 days after operation				
Absent	98	98	85	98
Present	2	2	2	2
Surgery related mortality				
Absent	100	100	87	100
Present	0	0	0	0

*Note*: Data are presented as numbers or median.

Abbreviations: C‐D, Clavien–Dindo classification; IQR, interquartile range.

The analysis of the conversion rate in the primary endpoint analysis population of 87 patients failed to meet the original statistical target of 94 cases, but the upper confidence limit of the exact confidence interval for the binomial distribution was 3.38, indicating that RAC was non‐inferior to the historical control of LC (*p* = 0.0006) (Table 5). Even if the excluded 13 patients would be added to the primary endpoint analysis, the non‐inferiority of RAC to the LC, which was the one of the secondary endpoints of this study, was proven (*p* = 0.0002) (Table S1).

**TABLE 5:** Conversion rate of the cohort for primary analysis (*N* = 87).

	*N*	%	90% confidence interval^a^		*p* Value^b,c^
			Lower	Upper	
Conversion					
Present	0	0	0	3.38	0.0006
Absent	87	100			

*Note*: Data are presented as numbers. (a) Accurate confidence interval on the binomial distribution (Clopper–Pearson). (b) Accurate one‐sided binomial test for null hypothesis P0 > 8.1% (non‐inferiority margin 2.7%) (Clopper–Pearson) (one‐sided *α* = 5%). (c) Judging it clinically insignificant when the upper limit of the confidence interval of the conversion rate of robotic‐assisted colectomy was 8.1% or higher.

### 3.3 Pathological findings

Pathological stages I, II, and III were found in 39%, 27%, and 34% of patients, respectively, and the proximal and distal margins were sufficiently estimated in all cases. One patient underwent surgery with combined resection of the abdominal wall and gonadal vessels due to invasions in these organs. Macroscopically unknown dissected margins (RX) were observed in two cases, and R0 resection was performed in 98% of patients (Table 6).

**TABLE 6:** Pathological findings.

	All patients		Patients for primary analysis	
	*N* = 100	%	*N* = 87	%
Macroscopic type				
0	23	23	20	23
1	10	10	7	8
2	63	63	58	67
3	3	3	1	1
4	0	0	0	0
5	1	1	1	1
Histology				
pap	2	2	2	2
tub	90	90	78	90
por	4	4	3	3.5
muc	3	3	3	3.5
others	1	1	1	1
Depth of invasion (pT)				
T1	25	25	21	24
T2	18	18	16	18
T3	43	43	38	44
T4	14	14	12	14
Lymph node metastasis (pN)				
N0	66	66	56	64
N1	29	29	26	30
N2	5	5	5	6
N3	0	0	0	0
Distant metastasis (sM)				
M0	100	100	87	100
M1	0	0	0	0
pStage				
I	39	39	33	38
II	27	27	23	26
III	34	34	31	36
IV	0	0	0	0
PM, cm (IQR)	13.9 (10.5–20.7)		14 (11–21.9)	
PMX	0	0	0	0
PM0	100	100	87	100
PM1	0	0	0	0
DM, cm (IQR)	14 (11–19)		14 (11–19.4)	
DMX	0	0	0	0
DM0	100	100	87	100
DM1	0	0	0	0
RM				
RMX	2	2	2	3
RM0	91	98	78	97
RM1	0	0	0	0
R0 resection				
RX	2	2	2	2
R0	98	98	85	98
R1/2	0	0	0	0

*Note*: Data are presented as numbers or median.

Abbreviation: IQR, interquartile range.

## 4 DISCUSSION

This study is the first multicenter prospective examination of the short‐term outcomes of robot‐assisted colectomy (RAC) for colonic cancer in Japan. The rate of conversion to laparotomy, which was the primary endpoint of this study, was 0%, indicating its non‐inferiority to the conversion rate in the historical control of LC based on previous reports, demonstrating the safety and feasibility of this surgical procedure.

To date, most reports on RAC have been based on retrospective studies. A randomized controlled study published in 2009 by Park et al.^19^ reported no difference in short‐ or long‐term outcomes between RAC and LC, although they analyzed a small sample size. Recent reports included studies based on relatively large databases and prospective studies. Schootman et al. compared data from the American College of Surgeons National Surgical Quality Improvement Program (2013–2015) on RAC (2233 cases) and LC (10 844 cases) with adjustment for selection bias based on propensity scores. The results of the study showed a lower conversion rate (5.7% vs. 18.6%, *p* < 0.05) and shorter length of hospital stay (5.1 vs. 5.3 days, *p* < 0.05) in the RAC group.^20^, ^21^ Kulaylat et al. also reported a significantly better conversion rate in RAC (6% in RAC vs. 11.5% in LC, *p* < 0.001), comparing 3864 cases of RAC to 40 063 cases of LC, using the same database and methods.^20^, ^21^ Furthermore, meta‐analyses reported by Ma et al. and Solaini et al.^22^, ^23^ suggested that right hemicolectomy (RAC) is an excellent tool for MIS, as it contributes to a reduced risk of conversion and earlier postoperative recovery. The analysis by Ma et al.^22^ showed a longer hospital stay in the laparoscopic group, lower complication rate in the robotic group, lower blood loss rate, shorter time to first flatus, and lower rate of conversion (odds ratio 0.34%, *p* = 0.008). Similarly, Solaini et al. showed a higher risk of conversion in LC (relative risk 1.7, *p* = 0.020) and a longer time to first flatus.^23^

JCOG0404, which was chosen as the historical control for this study, is a multicenter RCT that examined the non‐inferiority of OS of laparoscopic surgery to open surgery in patients with cStage II/III colon cancer. In this study, the short‐term surgical outcomes showed that laparoscopic surgery can be performed safely without increasing postoperative complications, with a longer operative time than open surgery, but better early recovery of bowel function, lower use of anesthetics, and shorter hospital stay. Regarding long‐term results, the primary endpoint of this study, the 5‐year survival rate was 90.4% in the open group and 91.8% in the laparoscopic group, which was comparable, but the statistical non‐inferiority of laparoscopy was not proven. However, the favorable results of this study make laparoscopic surgery an acceptable alternative.

Concerning the short‐term surgical outcomes of our study, no conversions or intraoperative adverse events occurred. The median blood loss was as low as 0 mL, and the rate of severe postoperative complications was as low as 4%, which compares favorably with previous reports. The median operative duration was 211 min, the time to first flatus after operation was 3 days, and the length of postoperative hospital stay was 6 days, similar to previous reports, including the JCOG0404 trial. With this procedure covered by insurance in Japan since April 2022, the number of medical facilities introducing this procedure is gradually increasing, and we believe that the results of this study positively support its further spread.

The true endpoint of surgery for malignant tumors is OS; however, a long period of observation is required before the results are obtained. Therefore, the rate of conversion, which can be evaluated in a short period of time and has been reported to be associated with postoperative complications, mortality, increased blood transfusions, and recurrence rate due to residual tumors, was set as the surrogate endpoint in this study.^24^, ^25^ Although several studies have shown a lower rate of conversion in RAC compared to LC, since RAC has just been introduced in Japan and is still in the learning curve stage, this study was designed as a non‐inferiority study to verify that the safety of the new technology is not inferior to that of established technologies.^23^, ^26^, ^27^, ^28^ Patients operated on by surgeons with less than three cases of experience in RAC were excluded from the population for the primary endpoint analysis. Therefore, the initially set number of 94 cases was not reached, and 87 cases were analyzed. However, because the null hypothesis was statistically rejected even in these 87 cases, we determined that the procedure's non‐inferiority was proven in this study.

Although patient registration for the study was completed by March 2022, follow‐up studies are ongoing to collect information on long‐term outcomes, including secondary endpoints such as OS and RFS, as well as the incidence of abdominal incisional hernia. The results are awaited to determine whether RAC has long‐term and oncological safety.

This study had some limitations. One was the insufficient number of cases in the cohort for primary endpoint analysis, as mentioned earlier. Furthermore, the criteria for the surgeons were that they were experts with sufficient experience in RAS for rectal cancer, but they had little experience in RAC. Although favorable outcomes were obtained for all surgeries, it may be difficult to generalize the results of this study to all institutions in Japan, where RAS is still in its early stages of widespread use. Moreover, information on cost was not collected in this study. The disadvantages of RAC are longer operative time and higher cost, but these may be overcome by shortening the duration of the operation and achieving a lower occurrence of complications through the skill enhancement of surgeons.^20^, ^21^ Novel research focusing on cost is expected in the future.

In conclusion, the rate of conversion to open surgery in robot‐assisted right hemicolectomy or right colectomy for colon cancer performed by expert surgeons was not inferior to that of LC. The favorable perioperative outcomes obtained with this procedure also demonstrate its safety and feasibility. Follow‐up studies are needed to validate the long‐term surgical outcomes, including the oncological safety of RAC.

## AUTHOR CONTRIBUTIONS

Shinichi Yamauchi, Akio Shiomi, Chu Matsuda, Ichiro Takemasa, Tsunekazu Hanai, Mamoru Uemura and Yusuke Kinugasa contributed to conceptualization, manuscript writing, and editing.

## FUNDING INFORMATION

This study was supported by funds from Intuitive Surgical Sàrl (Swiss Corporation).

## CONFLICT OF INTEREST STATEMENT

Yusuke Kinugasa received lecture fees from Intuitive Surgical G.K. All other authors have no conflict of interests to declare for this article. Ichiro Takemasa is an editorial member of *Annals of Gastroenterological Surgery*.

## ETHICAL STATEMENT

Approval of the research protocol: The protocol of this study was approved by the Tokyo Medical and Dental University Certified Review Board (approval number: NR2019‐001) and was in accordance with the ethical standards of the responsible committee on human experimentation and with the Helsinki Declaration of 1964 and later versions.

Informed Consent: Written informed consent was obtained from all patients.

Registry and the Registration No. of the study/trial: This study was registered in the Japan Registry of Clinical Trials (registration number: jRCT1032190036).

## Supporting information


Table S1.

## Data Availability

The data analyzed during the current study are available from the corresponding author on reasonable request.

## References

[1] WHO International agency for research on cancer. 2020. https://gco.iarc.fr/today/fact‐sheets‐cancers/9‐Rectum‐fact‐sheet.pdf

[2] Cancer Statistics in Japan. 2021. https://ganjoho.jp/reg_stat/statistics/data/dl/en.html/cancer_mortality(1958‐2019)E.xls

[3] Cancer Statistics in Japan. 2021. https://ganjoho.jp/reg_stat/statistics/data/dl/en.html/cancer_incidenceNCR(2016‐2018)E.xls

[4] Phillips EH , Franklin M , Carroll BJ , Fallas MJ , Ramos R , Rosenthal D . Laparoscopic colectomy. Ann Surg. 1992;216(6):703–7. 1466626 10.1097/00000658-199212000-00015 PMC1242720

[5] Lacy AM , Garcia‐Valdecasas JC , Delgado S , Castells A , Taura P , Pique JM , et al. Laparoscopy‐assisted colectomy versus open colectomy for treatment of non‐metastatic colon cancer: a randomised trial. Lancet. 2002;359(9325):2224–9. 12103285 10.1016/S0140-6736(02)09290-5

[6] Nelson H , Sargent D , Wieand HS , Fleshman J , Anvari M , Stryker SJ , et al. A comparison of laparoscopically assisted and open colectomy for colon cancer. N Engl J Med. 2004;350(20):2050–9. 15141043 10.1056/NEJMoa032651

[7] Bonjer HJ , Haglind E , Jeekel I , Kazemier G , Pahlman L , Hop WCJ , et al. Laparoscopic surgery versus open surgery for colon cancer: short‐term outcomes of a randomised trial. Lancet Oncol. 2005;6(7):477–84. 15992696 10.1016/S1470-2045(05)70221-7

[8] Guillou PJ , Quirke P , Thorpe H , Walker J , Jayne DG , Smith AM , et al. Short‐term endpoints of conventional versus laparoscopic‐assisted surgery in patients with colorectal cancer (MRC CLASICC trial): multicentre, randomised controlled trial. Lancet. 2005;365(9472):1718–26. 15894098 10.1016/S0140-6736(05)66545-2

[9] Jayne DG , Guillou PJ , Thorpe H , Quirke P , Copeland J , Smith AMH , et al. Randomized trial of laparoscopic‐assisted resection of colorectal carcinoma: 3‐year results of the UK MRC CLASICC trial group. J Clin Oncol. 2007;25(21):3061–8. 17634484 10.1200/JCO.2006.09.7758

[10] Colon Cancer Laparoscopic or Open Resection Study G , Buunen M , Veldkamp R , Hop WC , Kuhry E , Jeekel J , et al. Survival after laparoscopic surgery versus open surgery for colon cancer: long‐term outcome of a randomised clinical trial. Lancet Oncol. 2009;10(1):44–52. 19071061 10.1016/S1470-2045(08)70310-3

[11] Kitano S , Inomata M , Mizusawa J , Katayama H , Watanabe M , Yamamoto S , et al. Survival outcomes following laparoscopic versus open D3 dissection for stage II or III colon cancer (JCOG0404): a phase 3, randomised controlled trial. Lancet Gastroenterol Hepatol. 2017;2(4):261–8. 28404155 10.1016/S2468-1253(16)30207-2

[12] Rondelli F , Balzarotti R , Villa F , Guerra A , Avenia N , Mariani E , et al. Is robot‐assisted laparoscopic right colectomy more effective than the conventional laparoscopic procedure? A meta‐analysis of short‐term outcomes. Int J Surg. 2015;18:75–82. 25907328 10.1016/j.ijsu.2015.04.044

[13] Binder J , Kramer W . Robotically‐assisted laparoscopic radical prostatectomy. BJU Int. 2001;87(4):408–10. 11251539 10.1046/j.1464-410x.2001.00115.x

[14] Weber PA , Merola S , Wasielewski A , Ballantyne GH . Telerobotic‐assisted laparoscopic right and sigmoid colectomies for benign disease. Dis Colon Rectum. 2002;45(12):1689–94; discussion 95‐6. 12473897 10.1007/s10350-004-7261-2

[15] Yamaguchi T , Kinugasa Y , Shiomi A , Kagawa H , Yamakawa Y , Furuatni A , et al. Short‐ and long‐term outcomes of robotic‐assisted laparoscopic surgery for rectal cancer: results of a single high‐volume center in Japan. Int J Colorectal Dis. 2018;33(12):1755–62. 30191369 10.1007/s00384-018-3153-0

[16] Yamauchi S , Hanaoka M , Iwata N , Masuda T , Tokunaga M , Kinugasa Y . Robotic‐assisted surgery: expanding indication to colon cancer in Japan. J Anus Rectum Colon. 2022;6(2):77–82. 35572487 10.23922/jarc.2021-073 PMC9045855

[17] Yamamoto S , Inomata M , Katayama H , Mizusawa J , Etoh T , Konishi F , et al. Short‐term surgical outcomes from a randomized controlled trial to evaluate laparoscopic and open D3 dissection for stage II/III colon cancer: Japan clinical oncology group study JCOG 0404. Ann Surg. 2014;260(1):23–30. 24509190 10.1097/SLA.0000000000000499

[18] Nakajima K , Inomata M , Akagi T , Etoh T , Sugihara K , Watanabe M , et al. Quality control by photo documentation for evaluation of laparoscopic and open colectomy with D3 resection for stage II/III colorectal cancer: Japan clinical oncology group study JCOG 0404. Jpn J Clin Oncol. 2014;44(9):799–806. 25084776 10.1093/jjco/hyu083

[19] Park JS , Kang H , Park SY , Kim HJ , Woo IT , Park IK , et al. Long‐term oncologic after robotic versus laparoscopic right colectomy: a prospective randomized study. Surg Endosc. 2019;33(9):2975–81. 30456502 10.1007/s00464-018-6563-8

[20] Schootman M , Hendren S , Loux T , Ratnapradipa K , Eberth JM , Davidson NO . Differences in effectiveness and use of robotic surgery in patients undergoing minimally invasive colectomy. J Gastrointest Surg. 2017;21(8):1296–303. 28567574 10.1007/s11605-017-3460-8 PMC5576564

[21] Kulaylat AS , Mirkin KA , Puleo FJ , Hollenbeak CS , Messaris E . Robotic versus standard laparoscopic elective colectomy: where are the benefits? J Surg Res. 2018;224:72–8. 29506855 10.1016/j.jss.2017.11.059

[22] Ma S , Chen Y , Chen Y , Guo T , Yang X , Lu Y , et al. Short‐term outcomes of robotic‐assisted right colectomy compared with laparoscopic surgery: a systematic review and meta‐analysis. Asian J Surg. 2019;42(5):589–98. 30503268 10.1016/j.asjsur.2018.11.002

[23] Solaini L , Bazzocchi F , Cavaliere D , Avanzolini A , Cucchetti A , Ercolani G . Robotic versus laparoscopic right colectomy: an updated systematic review and meta‐analysis. Surg Endosc. 2018;32(3):1104–10. 29218671 10.1007/s00464-017-5980-4

[24] Marusch F , Gastinger I , Schneider C , Scheidbach H , Konradt J , Bruch HP , et al. Importance of conversion for results obtained with laparoscopic colorectal surgery. Dis Colon Rectum. 2001;44(2):207–14; discussion 14‐6. 11227937 10.1007/BF02234294

[25] Memon S , Heriot AG , Murphy DG , Bressel M , Lynch AC . Robotic versus laparoscopic proctectomy for rectal cancer: a meta‐analysis. Ann Surg Oncol. 2012;19(7):2095–101. 22350601 10.1245/s10434-012-2270-1

[26] Blumberg D . Robotic colectomy with intracorporeal anastomosis is feasible with no operative conversions during the learning curve for an experienced laparoscopic surgeon developing a robotics program. J Robot Surg. 2019;13(4):545–55. 30474786 10.1007/s11701-018-0895-1

[27] Scotton G , Contardo T , Zerbinati A , Tosato SM , Orsini C , Morpurgo E . From laparoscopic right colectomy with extracorporeal anastomosis to robot‐assisted intracorporeal anastomosis to totally robotic right colectomy for cancer: the evolution of robotic multiquadrant abdominal surgery. J Laparoendosc Adv Surg Tech A. 2018;28(10):1216–22. 30117748 10.1089/lap.2017.0693

[28] Lujan HJ , Plasencia G , Rivera BX , Molano A , Fagenson A , Jane LA , et al. Advantages of robotic right colectomy with Intracorporeal anastomosis. Surg Laparosc Endosc Percutan Tech. 2018;28(1):36–41. 28319493 10.1097/SLE.0000000000000384 PMC5802257

