# Supplementary material for: Robotic‐assisted colectomy for right‐sided colon cancer: Short‐term surgical outcomes of a multi‐institutional prospective cohort study in Japan
Source: Ann Gastroenterol Surg. 2023 May 23;7(6):932–9. doi: 10.1002/ags3.12694 (PMC10623957; doi:10.1002/ags3.12694)
Supplement: Supplementary file 1 — Table S1. [file AGS3-7-932-s001.docx]

Supplementary Table S1. Conversion rate of all patients (N=100)

|  |  | N | % | 90% confidence interval ^a)^ | | P value ^b)c)^ |
| --- | --- | --- | --- | --- | --- | --- |
|  |  |  |  | lower | upper |  |
| Conversion | |  |  |  |  |  |
|  | Present | 0 | 0 | 0 | 2.95 | 0.0002 |
|  | Absent | 100 | 100 |  |  |  |

Note: Data are presented as numbers.

a) accurate confidence interval on the binomial distribution (Clopper-Pearson)

b) accurate one-sided binomial test for null hypothesis P0>8.1% (non-inferiority margin 2.7%) (Clopper-Pearson) (one-sided α＝5%)

c) judging it clinically insignificant when the upper limit of the confidence interval of the conversion rate of robotic-assisted colectomy was 8.1% or higher.
